# Standard maximum likelihood analyses of alignments with gaps can be statistically inconsistent

**DOI:** 10.1371/currents.RRN1308

**Published:** 2012-03-13

**Authors:** Tandy Warnow

**Affiliations:** Professor of Computer Science, Department of Computer Science, University of Texas at Austin

## Abstract

Background

Most statistical methods for phylogenetic estimation in use today treat a gap (generally representing an insertion or deletion, i.e., indel) within the input sequence alignment as missing data. However, the statistical properties of this treatment of indels have not been fully investigated.

Results

We prove that maximum likelihood phylogeny estimation, treating indels as missing data, can be statistically inconsistent for a general (and rather simple) model of sequence evolution, even when given the true alignment. Therefore, accurate phylogeny estimation cannot be guaranteed for maximum likelihood analyses, even given arbitrarily long sequences, when indels are present and treated as missing data.

Conclusions

Our result shows that the standard statistical techniques used to estimate phylogenies from sequence alignments may have unfavorable statistical properties, even when the sequence alignment is accurate and the assumed substitution model matches the generation model. This suggests that the recent research focus on developing statistical methods that treat indel events properly is an important direction for phylogeny estimation.

## Background

### Phylogeny estimation from indel-free sequences

Theoretically, we now know a great deal about phylogenetic estimation.  We know, for example, that when sequences evolve with only substitutions (but no indels) under models such as the General Time Reversible (GTR) model, then accurate estimation of trees (with high probability) is guaranteed, provided that appropriate methods (such as maximum likelihood) are used and the sequences are “long enough” [1] [2] [3] [4] [5]. Simulation studies [3] [6] [7] have also shown that highly accurate trees can be estimated from sequences, especially when phylogenies are estimated using statistical methods (maximum likelihood or Bayesian methods, or some of the new "fast-converging" methods [1] [3] [5] [7]) that are based upon statistical models, such as GTR [8].  Excellent methods for maximum likelihood are available, with RAxML [9] and FastTree [10] probably among the most accurate for very large-scale analyses (FastTree is less well known than RAxML, however, as shown in [11], FastTree analyses are nearly as accurate as RAxML and run in a fraction of the time).

However, sequence evolution includes indels, and so typically a phylogenetic analysis begins with a set of sequences of different lengths.  A phylogenetic analysis of such a dataset, therefore, must either first align these sequences before applying a phylogenetic estimation method or it must co-estimate the alignment and tree at the same time.  (The third approach estimates the tree without ever estimating an alignment, but is much less in vogue.)

In this paper, we discuss theoretical and empirical aspects of phylogeny estimation in the presence of indels, focusing on two-phase methods using maximum likelihood (henceforth referred to as "ML").  We include, for completeness sake, a discussion of co-estimation methods. 

### Co-estimation methods

Methods that estimate trees and alignments from unaligned sequences (i.e., co-estimation methods) have also been developed, including POY [12], SATé [13] [14] [15] and SATCHMO [16]. POY is a heuristic for a maximum parsimony analysis in which gaps are penalized, based upon a gap-open cost and a gap-extend cost.  The performance of POY is quite controversial, however, with some studies suggesting that trees and alignments estimated by POY are less accurate than standard two-phase methods (see, for example,  [17] [18]) while other studies show that the performance depends substantially on the gap penalty [19] [20]. Also, because POY is based upon parsimony, it is not statistically consistent. SATé uses an iterative technique, with each iteration computing a new alignment and then estimating a GTR ML tree on the alignment.  Analyses on both biological and simulated data [13] [14] show that SATé produces more accurate trees and alignments than the leading two-phase methods, especially on datasets with thousands of sequences; however, SATé also has no statistical guarantees [13]. Finally, SATCHMO-JS is another co-estimation method without any statistical guarantees [16], but which has also been shown to produce improved alignments and trees; however, it is only applicable to amino-acid alignment and phylogeny estimation. Statistically-based methods that co-estimate trees and alignments with reference to a stochastic model of evolution that includes indels as well as substitutions have also been developed [21] [22] [23] [24] [25] [26] [27] [28] [29]. However, all of these are computationally very intensive. In fact, of these, only BAli-Phy [21] [22] [23] is able to run on datasets with 100 sequences (but not necessarily larger). Thus, no co-estimation methods with statistical guarantees can be run on datasets of the size that would be most common among Tree of Life projects.

## Two-phase methods

Despite the progress in co-estimation methods, most phylogenies are estimated in two phases: first an alignment is estimated, and then a tree is computed on the alignment. Once the sequences are aligned, a decision must be made about how to  treat the gaps in the alignment. In current practice, the following are the dominant gap-treatments:

    • Remove all sites in which any gap appears, thus reducing to a gap-free alignment with fewer sites, 

    • Assign an additional “fictitious” state for each gap, 

    • Code all the gaps in the alignment, and treat the presence/absence of gaps as a binary character (complementing the original sequence alignment character data), and

    • Treat the gaps as missing data. In parsimony analyses, this is often treated by finding the best nucleotide to replace the gap, but in likelihood-based analyses, this is often treated by summing the likelihood over all possible nucleotides for each gap.

The first option of removing all sites with gaps has the advantage of being statistically consistent for models in which the substitution process and the mechanism producing  insertions and deletions are independent, but it has the disadvantage of removing data – and could result in sequence alignments that have so few sites as to be phylogenetically  uninformative. Indeed, while this may not happen on small datasets, on large nucleotide datasets, this could lead to empty alignments.

The second option of assigning an additional state for each gap presents other challenges. By definition, the true alignment represents positional homology, and hence two positions that have a nucleotide in a site constrain all nodes on the evolutionary path between them to also have a nucleotide in that position. In other words, ensuring that the model makes phylogenetic sense is rather complicated. Therefore, the substitution process must be extended carefully to handle an additional fictitious state properly. Finally, when the indel process can insert and delete several nucleotides at a time, the sites within the alignment no longer evolve independently, making this treatment invalid. 

The third option, of coding each gap (maximal contiguous collection of dashes) in the alignment, includes a collection of techniques, ranging  from extremely simple (create a single binary presence/absence character for each position that contains any gap) to very complex techniques. Software to automatically produce these additional binary characters encoding the gaps in a given alignment includes GapCoder [30] and also software developed for complex indel coding by Muller [31]. Simulation studies have shown improvements in tree estimation obtained through gap-coding over treating gaps as missing data (e.g., [32] [33]). However, the use of gap-coding is controversial (see the discussion in [32]), and not the dominant technique in phylogenetic analyses. Instead, the most frequently used option (and the default for most software) is to treat gaps as missing data. Because of this, we focus our discussion on the impact of treating gaps as missing data in phylogenetic analyses based upon ML.

## Jukes-Cantor Model of DNA Sequence Evolution

We begin with the Jukes-Cantor (JC) model of DNA sequence evolution [34], a special case of the more commonly used General Time Reversible (GTR) model.  The JC model of site evolution assumes that only substitutions occur, and is characterized by a pair of parameters (T,θ), where T is a rooted binary tree with leaves labeled by a set S of taxa, and θ is a set of edge substitution probabilities, p(e) (one for every edge e ∈ E(T)). Each substitution probability p(e) is constrained to satisfy 0 < p(e) < 3/4, and and gives the probability that the site changes on the edge e. The nucleotide at the root of the tree is selected from the uniform distribution over {A,C,T,G}. If the site changes its nucleotide state on edge e, then it changes with equal probability to one of the remaining three states. To use JC for modeling sequences, we assume that all sites evolve independently and identically (i.i.d). Note, therefore, that the JC model does not incorporate any mechanism for the formation of indels, so that sequences that are generated by this model will never have gaps.

## Maximum likelihood on gap-free alignments

Letting the tree topology T and alignment A be fixed, we define ML_JC_ (A,T) := sup_θ_ P(A|(T,θ)). That is, ML_JC_(A,T) is the supremum of all likelihood scores obtained for JC model trees with the same fixed tree topology T (but allowing θ to vary). Although the likelihood is continuous, the supremum may not actually be achieved for some θ because the range of values allowed for this parameter is not a closed set; that is, the supremum may be approached by parameter values θ for which some of the p(e) are arbitrarily close to the boundary values 0 or 3/4. Finally, we can talk about the JC ML tree for a fixed gap-free alignment A, as the tree T such that the likelihood ML_JC_(A,T) is maximized over all trees.

Maximum likelihood (ML) inference of the parameter T under the JC model is defined as follows:

    • Input: sequence alignment A containing no gaps

    • Output: all model trees T such that ML_JC_(A,T) is maximized

Algorithmically, ML phylogeny estimation typically operates by searching through the space of all binary trees, estimating  parameters of the model (in this case, the branch substitution parameters) on each tree so as to maximize likelihood on that tree, and then returns the tree and its model parameters that return the highest likelihood.  

## Maximum likelihood analysis on alignments with gaps

We now discuss ML analysis when the input sequence alignment contains gaps, and gaps are treated as missing data.  The same algorithmic approach is used as when the input alignment does not contain gaps, except that the likelihood calculation must also be able to work with gapped sequences. As discussed above, there are several different ways of treating gaps, but the standard technique treats gaps as missing data.   

## Theoretical results when treating gaps as missing data

It is well known that ML is statistically consistent for the GTR model (and hence for its submodels, such as Jukes-Cantor), when the data are generated by the GTR model and the optimization problem is solved exactly. However, we will show that ML, treating gaps as missing data,  can be inconsistent under these conditions, when the input sequence alignments contain gaps. In other words, we will prove that ML can produce the wrong tree, under some conditions on the input sequence alignment.  

Let S be a set of DNA sequences in an alignment A. We will say that the alignment A is "monotypic" if for each site in A, there is exactly one nucleotide type (that is, all A’s, all C’s, all T’s, or all G’s). In particular, we do not allow any site to be entirely gapped. For example, the following is a monotypic alignment:

    s1 =  A − −

    s2 =  − C −

    s3 =  A − −

    s4 =  − − −

    s5 =  − − −

    s6 =  − − T

    s7=  A − −

The following results were established in [13]:

Lemma 1. Let A be a monotypic alignment for the set S of sequences, and let T be an arbitrary tree on S. If gaps are treated as missing data, then ML_JC_(A,T) =(1/4)^R^, where R is the number of sites in the alignment A.

Proof. This result follows from Lemma 1 in [13], but we sketch the proof here. For any tree T, the optimal settings of the edge substitution parameters on T have p(e)=0 for all edges (more correctly, the supremum ML_JC_ (A,T) is realized by a sequence of parameter values in which all the p(e) converge to 0). For this setting of the substitution parameters, the probability of the data is just the probability of picking the correct state for that site, which is 1/4 under the JC model. Hence, the ML score of the alignment, given the tree T, is (1/4)^R^, where R is the number of sites in A.

Theorem 1. Let A be a monotypic alignment for set S. Then all trees on S are optimal solutions for ML under Jukes-Cantor, if gaps are treated as missing data.

Proof. This result follows from Theorem 2 in [13], but we sketch the proof here. By Lemma 1, for monotypic alignments A, the JC ML scores for any tree are the same, so all trees are optimal solutions for ML under JC.

This theorem indicates a potential problem with treating gaps as missing data. If the mechanism generating the data has a high probability of producing aligned sequences that are monotypic for some parameter values, then it will be difficult to reliably infer the underlying phylogenetic tree if the gaps are treated merely as missing data rather than features of the data  that are informative about the path that evolution has taken. More specifically, for those models of evolution for which monotypic alignments have non-zero probability, ML, treating gaps as missing data, may not be statistically consistent.

## Discussion 

Theorem 1 shows that treating gaps as missing data has the potential to result in meaningless phylogenetic estimations, even when analyzed under maximum likelihood (ML) for the correct model, since - under an extreme case in which the substitution probabilities are all zero - all trees are equally good solutions to maximum likelihood.  In other words, what this theoretical result shows is that under an extreme condition in which substitution probabilities are zero, *treating gaps as missing data in a ML analysis is statistically inconsistent.*

We now compare this observation from [35], showing that the indel process itself can contain sufficient information to identify the tree topology. In other words, it is possible, theoretically, to estimate the tree topology perfectly even from monotypic alignments, provided that the indels are *not* treated as missing data. Therefore, monotypic alignments are *not* phylogenetically uninformative if the alignments contain indels. This should be contrasted to monotypic alignments without indels: these *are* phylogenetically uninformative because no changes of any type -- substitution or indel - occur on any branch of the tree.  In other words, the usual way of thinking about sequence evolution in which sites evolve only with substitutions would lead one to think of monotypic alignments as arising only when all branch lengths are zero.  This is not the case when indels as well as substitutions can occur since they each have their own evolutionary parameters, because then evolution can occur with indels but without substitutions, and produce monotypic alignments.  As this discussion shows, such model conditions are still identifiable because of the results from [35], but treating gaps as missing data will fail to be statistically consistent.  Thus, there *is* phylogenetic signal in an alignment that contains gaps even for the case of monotypic alignments, and this signal *can* be used to estimate the true tree, provided that appropriate methods are used.  The point to be taken is that treating gaps as missing data within a standard ML analysis is *not *an appropriate method. 

In contrast to this theoretical result, we consider the performance in practice of statistically-based methods such as ML and Bayesian MCMC. Simulation studies when sequences evolve without indels have shown that these statistically-based methods produce highly accurate trees, typically better than trees estimated using maximum parsimony or distance-based methods (see, for example, [36]). Similarly, simulation studies when sequences evolve with indels as well as substitutions have shown that ML and Bayesian MCMC produce trees that are typically more accurate than trees estimated using maximum parsimony of distance-based methods [8], provided that they are used with highly accurate alignments. On the other hand, these studies have also shown that alignment error has a very large impact on phylogenetic tree estimation error, especially for large datasets that have evolved with high rates of evolution [8] [13] [14] [37], and that reasonably accurate estimation of alignments of very large datasets (with upwards of a few thousand sequences) is extremely difficult [37].

Of particular interest here, however, is the observation in these studies that even when analyzing the true alignment, the error rate for ML trees increases with the rate of evolution (see, for example, [8] [13] [14]). While some of this increase is likely due to the increase in the substitution rate, it is possible that some of this increased error is due to the failure to properly use the indels within the true alignment. Therefore, the high degree of confidence that the systematics community has in these statistical methods should be attenuated slightly, at least when analyzing alignments with moderate to high numbers of indels. Under these conditions, the failure of standard ML and Bayesian MCMC methods to use the gaps in a statistically rigorous way could be substantial.

The  theorem in this paper holds under the assumption of all substitution probabilities being zero (the case where only indels but no substitutions occur).  Thus, this theoretical result can be criticized as being applicable only to a biologically unrealistic case.   A careful reader could therefore ask "Is maximum likelihood, treating gaps as missing data, provably statistically consistent for model conditions with substitutions and not just indels?" The answer is that there are no published theoretical results establishing statistical consistency for maximum likelihood when gaps are treated as missing data.  However, as was shown in [35], statistically consistent estimation of trees *is *possible if gaps are treated properly (not as missing data), and it is certainly possible for other methods to be developed that would be statistically consistent.  The point we make here is only that the standard ML analysis, treating gaps as missing data, does not have that guarantee.

## Conclusions

These results add to the growing literature about theoretical guarantees (or lack thereof) in phylogenetic analysis. Unfortunately, what we now know is that theoretical guarantees for phylogeny estimation have only been established for very restrictive conditions: indel-free evolution (so that alignment is not an issue) with well-behaved site substitution models. This raises the real possibility that the standard likelihood-based methods of analysis (e.g., MrBayes [38], RAxML [9], PAUP* [39], PhyML [36], FastTree [10], etc.) may not be statistically consistent (even on the true alignment!) when sequences evolve with indels.  

From an empirical viewpoint, multiple sequence alignment estimation on nucleotide datasets is difficult, especially on large datasets [37], and poor alignment estimation results in inaccurate trees [8] [13] [14] [37]. SATé has been shown to be able to co-estimate highly accurate alignments and trees, and does so very quickly, even on datasets with thousands of sequences. However, SATé has no statistical guarantees.  In contrast, methods with statistical guarantees, like BAli-Phy, are computationally infeasible for Tree of Life projects.   

Clearly, what is needed is the creation of statistically-based methods that treat both indels and substitutions in a statistically consistent manner, and that can run on large datasets (with at least hundreds but preferably thousands of sequences). Guarantees of statistical consistency do not necessarily yield good performance in practice, but they can lead to methods with good empirical performance (and have the potential to produce much more accurate results than statistically inconsistent methods). Therefore, an effort should be made to develop such methods, and to test these methods on both biological and simulated data, in order to evaluate their accuracy under realistic conditions. Until then, phylogenetic analyses can certainly be based upon standard two-phase approaches, but biologists should use these standard methods with caution - realizing that even the best of the current two-phase methods do not have statistical guarantees.

## Acknowledgements

The author thanks Steve N. Evans for stimulating and helpful discussions, and the two anonymous referees for their comments.

## Funding information

This research was supported by two grants from the NSF (ATOL grant DEB 0733029 [40] and DBI 1062335), a John Simon Guggenheim Memorial Foundation Fellowship, and a David Bruton Jr. Centennial Professorship. 

## Competing interests

The author has declared that no competing interests exist.
